# A Mechanistic View of the Light-Induced Synthesis of Silver Nanoparticles Using Extracellular Polymeric Substances of *Chlamydomonas reinhardtii*

**DOI:** 10.3390/molecules24193506

**Published:** 2019-09-27

**Authors:** Ashiqur Rahman, Shishir Kumar, Adarsh Bafana, Julia Lin, Si Amar Dahoumane, Clayton Jeffryes

**Affiliations:** 1 Nanobiomaterials and Bioprocessing Laboratory (NABLAB), Dan F. Smith Department of Chemical Engineering, Lamar University, Beaumont, TX 77710, USA; arahman2@lamar.edu (A.R.); skumar1@lamar.edu (S.K.); abafana@lamar.edu (A.B.); jlin@lamar.edu (J.L.); 2 School of Biological Sciences and Engineering, Yachay Tech University, Hacienda San José s/n, San Miguel de Urcuquí 100119, Ecuador; sdahoumane@yachaytech.edu.ec; 3 Center for Advances in Water & Air Quality, Lamar University, 211 Redbird Ln, Box 10888, Beaumont, TX 77710-0088, USA

**Keywords:** EPS, nanobiomaterials, bottom-up, AgNPs, algal synthesis, factorial design, photon

## Abstract

In the current study, extracellular polymeric substances (EPS) of *Chlamydomonas reinhardtii* and photon energy biosynthetically converted Ag^+^ to silver nanoparticles (AgNPs). The reaction mechanism began with the non-photon-dependent adsorption of Ag^+^ to EPS biomolecules. An electron from the EPS biomolecules was then donated to reduce Ag^+^ to Ag^0^, while a simultaneous release of H^+^ acidified the reaction mixture. The acidification of the media and production rate of AgNPs increased with increasing light intensity, indicating the light-dependent nature of the AgNP synthesis process. In addition, the extent of Ag^+^ disappearance from the aqueous phase and the AgNP production rate were both dependent on the quantity of EPS in the reaction mixture, indicating Ag^+^ adsorption to EPS as an important step in AgNP production. Following the reaction, stabilization of the NPs took place as a function of EPS concentration. The shifts in the intensities and positions of the functional groups, detected by Fourier-transform infrared spectroscopy (FTIR), indicated the potential functional groups in the EPS that reduced Ag^+^, capped Ag^0^, and produced stable AgNPs. Based on these findings, a hypothetic three-step, EPS-mediated biosynthesis mechanism, which includes a light-independent adsorption of Ag^+^, a light-dependent reduction of Ag^+^ to Ag^0^, and an EPS concentration-dependent stabilization of Ag^0^ to AgNPs, has been proposed.

## 1. Introduction

The synthesis of silver nanoparticles (AgNPs) via various green methods has been widely explored over the last few years as a more sustainable alternative to traditional synthesis methods [1,2,3,4,5,6]. Among the green methods, biosynthetic conversion of Ag^+^ to AgNPs using algae has gained much attention among researchers [7,8,9,10]. Various biomolecules present in both living cultures and cell culture supernatants of algae have been reported to catalyze these reactions [11,12,13]. For example, proteins extracted from *Chlorella vulgaris* promoted AgNP formation through hydroxyl and carboxyl functional groups [14]. Among the recent studies, Arévalo-Gallegos et al. used *Botrryococcus braunii*, a green microalga, and found that various functional groups, including carbonyl, hydroxyl, and amide, were responsible for the synthesis of AgNPs [7]. The same group also found that photons facilitated both the extra- and intra-cellular synthesis of AgNPs. While it is well established that photon input is necessary for the biosynthesis of AgNPs [11,15,16,17], a constitutive relationship between photons and NP production rates and yields has yet to be explored.

In the current study, we used extracellular polymeric substances (EPS) from the freshwater microalga *Chlamydomonas reinhardtii* to biosynthesize AgNPs. A constitutive relationship between the photon input, EPS concentration, and biosynthesis of AgNPs was studied to identify a possible underlying mechanism. Experiments were designed using a one-factor-at-a-time (OFAT) method to measure the sole impact of light on the process. In addition, a factorial experiment was implemented to study the impact of varying both photon input and EPS concentration. Experiments were carried out to measure Ag^+^ adsorption to the EPS and to determine any light-independent processes. Based on the observations made in this study, a three-step synthesis mechanism, which includes a light-independent adsorption, a light-dependent reduction, and an EPS-concentration-dependent stabilization, is proposed. Although a few mechanisms have been proposed in literature [18,19,20], they have focused primarily on molecular-scale reaction mechanisms. The current work analyzed how to quantify NP production based on process inputs. To the best of our knowledge, this is the first attempt to relate aqueous Ag^+^ concentration, photon input, and EPS concentration to AgNP production. This study represents an advance toward the goal of modeling and developing scalable photobioreactor (PBR) processes for metal NP production. In addition, this study could enable future investigations on the sorption capacity of the EPS from *C. reinhardtii* toward capturing metal cations, leading towards the application of an EPS–PBR–wastewater system [21,22].

## 2. Results and Discussion

### 2.1. Light-Independent Adsorption of Ag^+^ by EPS

**Figure 1:**
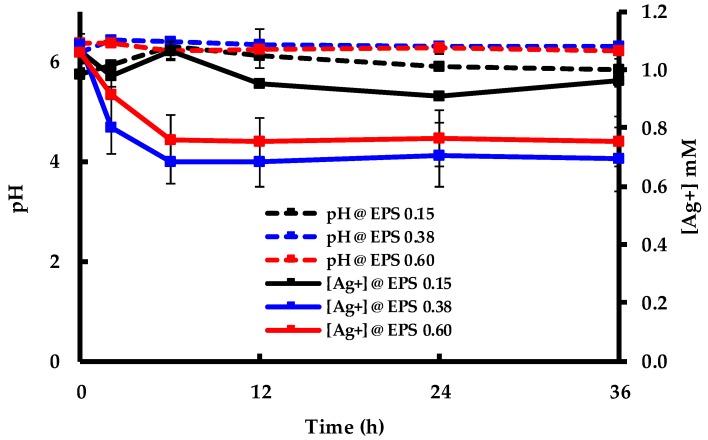
Change in pH and [Ag^+^] after the addition of AgNO_3_ to extracellular polymeric substances (EPS) solutions in the dark.

The adsorption of Ag^+^ to EPS at 0.15, 0.38, and 0.60 mg mL^−1^ EPS in the dark was measured in triplicate, and the results are presented in Figure 1. The figure shows the change of pH and [Ag^+^] over the first 36 h of the experiment. Although no significant change in pH was observed, there was an obvious disappearance of Ag^+^ in the liquid phase. The equilibrium concentration was reached within 6 hours, and Ag^+^ concentration remained constant for the remainder of the experiment. Additionally, more Ag^+^ was removed from the liquid phase as the EPS concentration increased. These results indicate that Ag^+^ adsorbs to EPS in the dark, without the need of photon energy. The stable pH, as shown in Figure 1, indicates that there was no conversion of Ag^+^ to Ag^0^, which is known to produce H^+^ and therefore lower the pH [17]. In conjunction with the data presented in the following sections, this suggests that Ag^+^ adsorption to EPS is the first step of the EPS-mediated AgNP biosynthesis process and can explain the subsequent steps of the reaction mechanism.

### 2.2. Conversion of Ag^+^ to AgNPs Using Photon Energy

**Figure 2:**
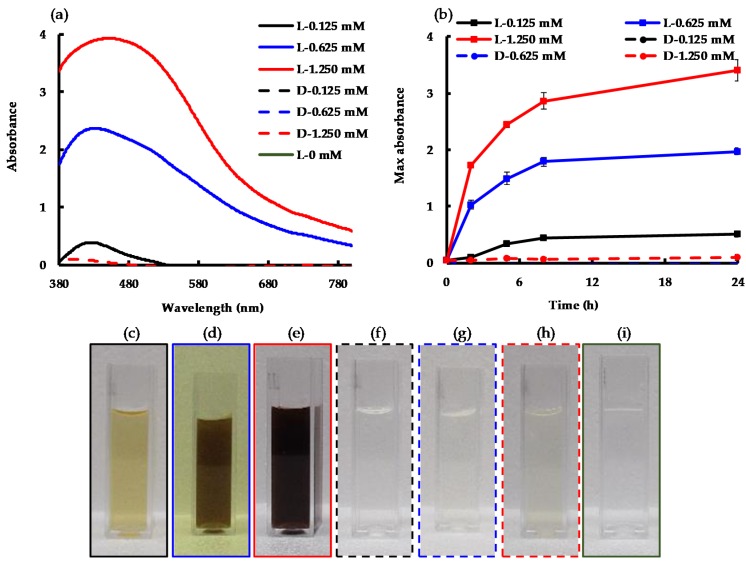
(**a**) Spectrophotometric measurements at 24 h; (**b**) Change of silver nanoparticle (AgNP) surface plasmon resonance (SPR) intensity vs. time over 24 h; (**c**–**i**) Reaction mixture in 1 cm pathlength cuvettes after 24 h of synthesis (**c**) L-0.125 mM; (**d**) L-0.625 mM; (**e**) L-1.250 mM; (**f**) D-0.125 mM (**g**) D-0.625 mM; (**h**) D-1.250 mM; (**i**) L-0 mM. “L-” indicates “in the light”; “D-” indicates “in the dark”; the values following are the concentrations of Ag^+^ in the synthesis reaction. The border of each photo (**c**–**i**) matches the series lines in (**a**,**b**).

UV–Visible spectra, recorded 24 h after adding different concentrations of AgNO_3_ to 0.46 mg mL^−1^ EPS from *C. reinhardtii* culture supernatant, are presented in Figure 2a. Samples that were exposed to light displayed a surface plasmon resonance (SPR) band between 425 nm and 480 nm due to the collective oscillation of the surface electrons of metallic silver, indicating the formation of AgNPs [23]. L-1.250 mM (where “L” stands for “exposed to light” and 1.250 mM indicates the initial AgNO_3_ concentration in the synthesis reaction), showed an SPR maximum after 24 h. In contrast, samples kept in the dark (represented by sample names beginning with “D”) did not exhibit any SPR absorption band, so the corresponding data series were indiscernible from the x-axis (Figure 2a,b). This established the necessity of photon input for the synthesis of AgNPs from Ag^+^ in the presence of *C. reinhardtii* EPS. On the other hand, the EPS-only control sample did not show any SPR absorption band, as can be seen by the L-0 mM sample (Figure 2a), verifying that the only SPR bands observed in these systems came from the conversion of Ag^+^ to AgNPs in the presence of EPS and under light exposure.

The change in the SPR band intensity measured with respect to time at the maximum absorption wavelength is presented in Figure 2b. The results show little to no visible SPR absorption band for the experiments conducted in the dark (dashed lines) when compared to similar experiments carried out in the light (solid lines). A higher maximum absorption of AgNPs, which was reached at 24 h, was observed with higher AgNO_3_ concentrations. This difference was also observed visually by the increasing intensity of the brown color as the Ag^+^ input increased, corresponding to increased AgNP production (Figure 2c–e). On the other hand, the samples kept in the dark at the three different AgNO_3_ concentrations (Figure 2f–h) appeared similar to the sample without AgNO_3_ (Figure 2i), as there was no formation of AgNPs in the dark. Furthermore, increasing the Ag^+^ input from 0.625 mM to 1.250 mM did not double the SPR absorbance, increasing it by only 73%. Likewise, increasing the Ag^+^ input 10-fold, from 0.125 mM to 1.250 mM, increased the SPR absorbance by only 6-fold. In the current experiment, we used a fixed concentration of EPS (i.e., 0.46 mg mL^−1^ EPS from *C. reinhardtii* culture supernatant) and measured the absorbance of stable AgNPs. Therefore, the current results indicate the possibility of aggregation of unstable or partially stable AgNPs as we increased the Ag^+^/EPS ratio. It is well-established that EPS work as a stabilizing agent to cap AgNPs and prevent their aggregation [14,24].

**Figure 3:**
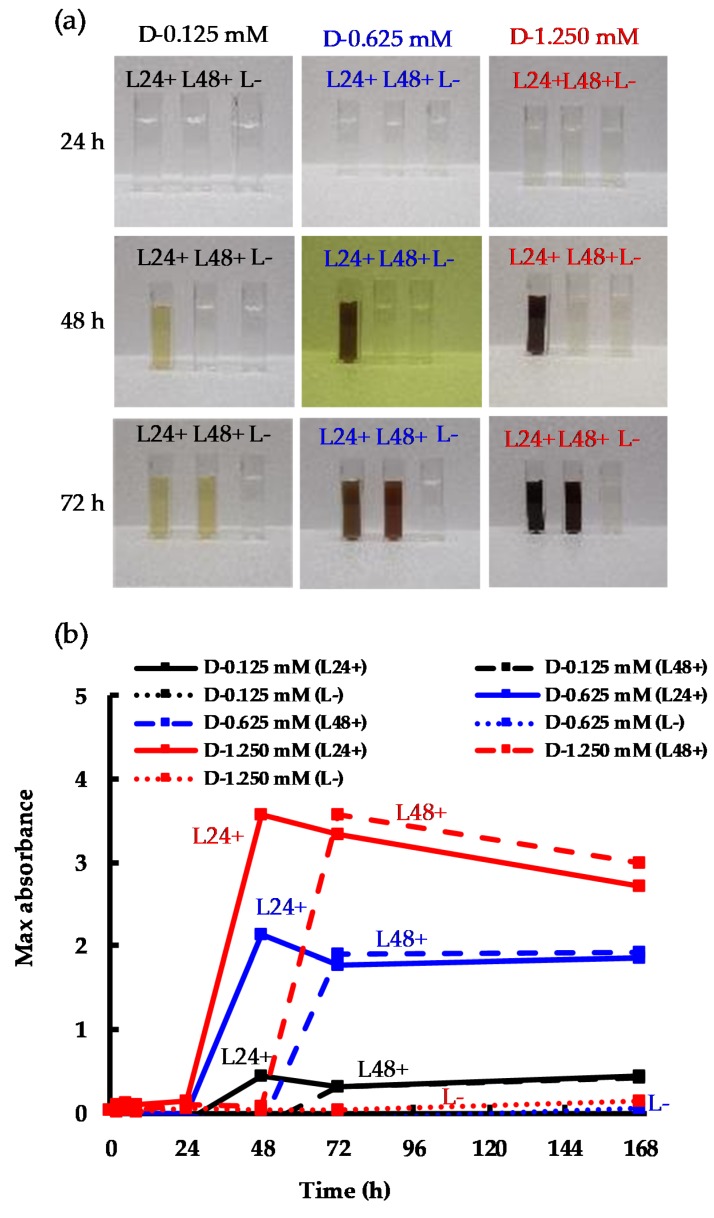
Experiments initially kept in the dark and then exposed to light after 24 h and 48 h: (**a**) change in color; (**b**) change of AgNP SPR intensity vs. time over 168 h.

To further evaluate the impact of light on AgNP biosynthesis, an OFAT experimental design was used. Each experiment was carried out in triplicate. All three samples remained in the dark for the first 24 h. After 24 h, one replicate was exposed to light (L24+), and after 48 hours the second replicate was also exposed to light (L48+). The third replicate (L-) was kept in the dark for the duration of the experiment as a negative control (the method used is presented in detail in the Materials and Methods section). The characteristic color changes of the L24+ and L48+ solutions after their respective exposure to light (Figure 3), from transparent to dark brown, indicated the synthesis of AgNPs (Figure 3a). In contrast, the replicate that was not exposed to light (L-) showed no change in color. Interestingly, the EPS maintained their reductive capabilities in the dark, but required light to promote the production of AgNPs. This can be seen in Figure 3b, where L24+ and L48+ had similar patterns and plateaued at the same value. Although higher SPR absorbances were recorded at 1.250 mM AgNO_3_ than at 0.625 mM, the increase was only 67% for L24+ samples and 87% for the L48+, a similar result to that shown in Figure 2b. These results corroborated our previous findings that demonstrated that the yield of AgNP production was not complete [13,25], and might be attributed to two causes: (i) the EPS reaching their maximum reduction capability as the Ag^+^/EPS ratio increased and (ii) a fraction of the as-produced AgNPs undergoing sedimentation, as mentioned in Figure 2b. Nevertheless, the current results demonstrate that a source of energy (i.e., photons) is an obligate requirement to enable the conversion of Ag^+^ to Ag^0^, while EPS concentration dominates the stabilization of Ag^0^ to yield stable AgNPs.

### 2.3. Combined Impact of Light and EPS on AgNP Production and Stabilization

**Figure 4:**
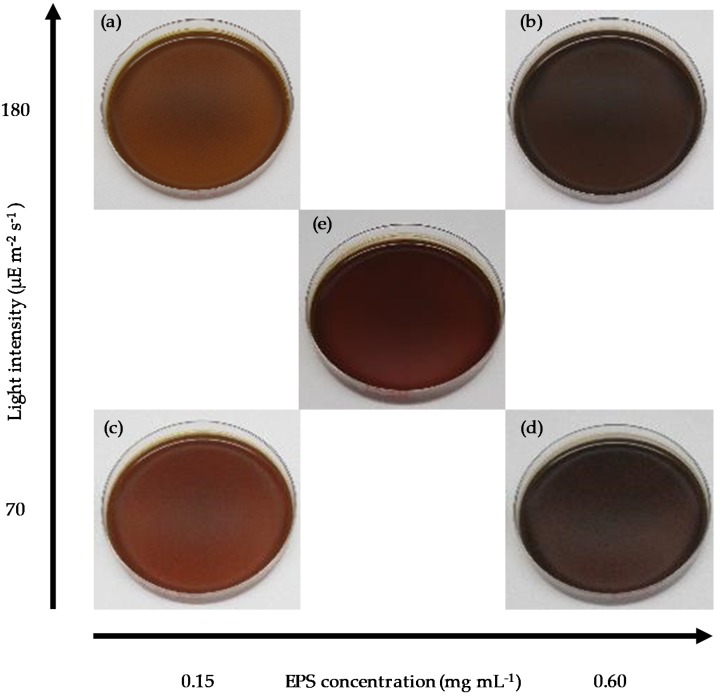
Digital images of samples: (**a**) EPS-0.15-1 mM-180 μE; (**b**) EPS-0.60-1 mM-180 μE; (**c**) EPS-0.15-1 mM-70 μE; (**d**) EPS-0.60-1 mM-70 μE; (**e**) EPS-0.38-1 mM-125 μE.

**Figure 5:**
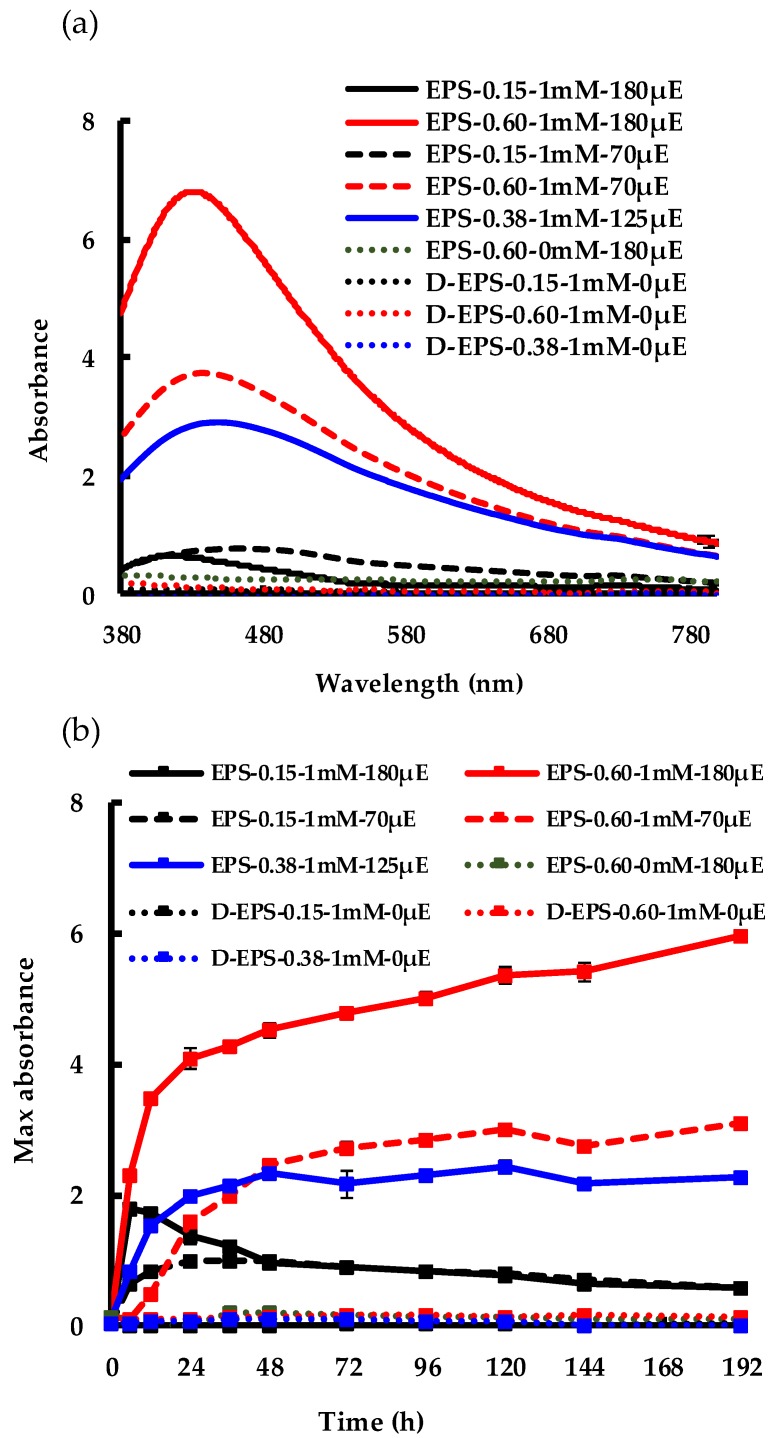
AgNP synthesis at different light intensities and EPS concentrations: (**a**) spectrophotometric measurements at 192 h; (**b**) change of SPR intensity vs. time over 192 h.

**Figure 6:**
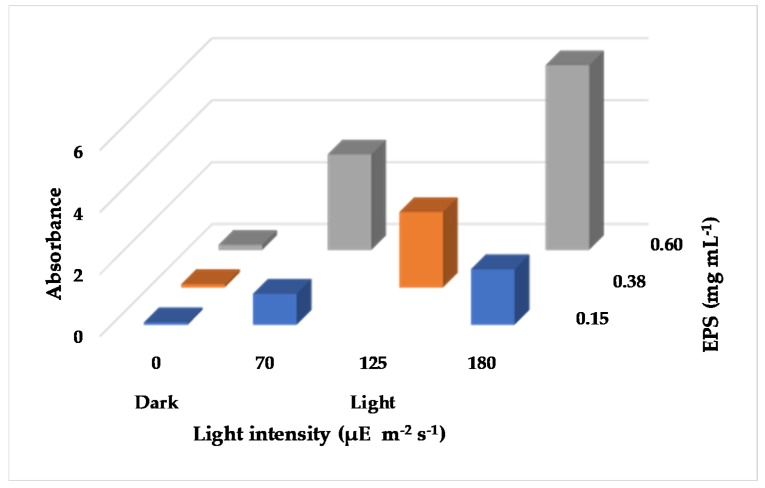
3D bar graph showing the maximum SPR absorbance at various light intensities and EPS concentrations.

To further investigate the impact of light on the AgNP biosynthesis process, experiments were carried out over a range of purified EPS concentrations in combination with varying light intensities. Figure 4 and Figure 5 show the results from a 2^2^ (two level two factor) factorial design experiment (details are provided in the Materials and Methods section). The images presented in Figure 4 visually confirmed a greater formation of AgNPs at increased EPS concentration and light intensity, while the AgNO_3_ concentration was constant (1 mM). In addition, Figure 5a shows the AgNP SPR band at wavelengths ranging from 425 nm to 480 nm at 192 h after Ag^+^ addition. The highest SPR absorbance was obtained from the EPS-0.60-1 mM-180 μE sample, the synthesis reaction with the highest input of light and EPS, corroborating the macroscopic aspect of Figure 4. On the other hand, when the amount of EPS or light intensity was decreased, the intensity of the SPR band decreased. Samples kept in the dark exhibited a very weak to no SPR band, regardless of the EPS concentration. Figure 5b shows the change of the SPR intensity vs. time over 192 h, measured at the maximum absorbance wavelength. It is noticeable that the SPR intensity increased as both the light intensity and EPS concentration increased. However, at low EPS concentration and high light intensity, the particle did not stabilize, as was seen by an increase in the AgNP SPR band in the first 6 hours and a ~67% decrease over time. As EPS concentration increased, there was a ~93% increase in AgNP production. These results indicate that both EPS concentration and light intensity are limiting variables for the biosynthesis process, because they are both necessary to promote the synthesis of stable AgNPs. This finding agrees with the results obtained from the OFAT experiment (Figure 1 and Figure 2). The maximum synthesis of AgNPs took place at maximum light intensity and EPS concentration, as depicted in the 3D bar graph in Figure 6. The data series of the dark control experiments stayed close to the x-axis and did not show any SPR absorption bands. Additionally, EPS in light without Ag^+^ (i.e., EPS-0.60-0 mM-180 μE sample in Figure 5a,b) showed no absorption. These results indicate the conversion of Ag^+^ to Ag^0^ and AgNPs by both light and EPS, followed by AgNP stabilization by EPS. Overall, the current findings suggest that the initial increase in SPR absorbance is related to the light intensity, while the final yield is related to the EPS concentration. This information could be exploited to design further experiments that will determine the rate limiting substrate, and therefore will predict the kinetics and yield of the biosynthesis process, paving the way to scalable, permanent PBR systems.

**Figure 7:**
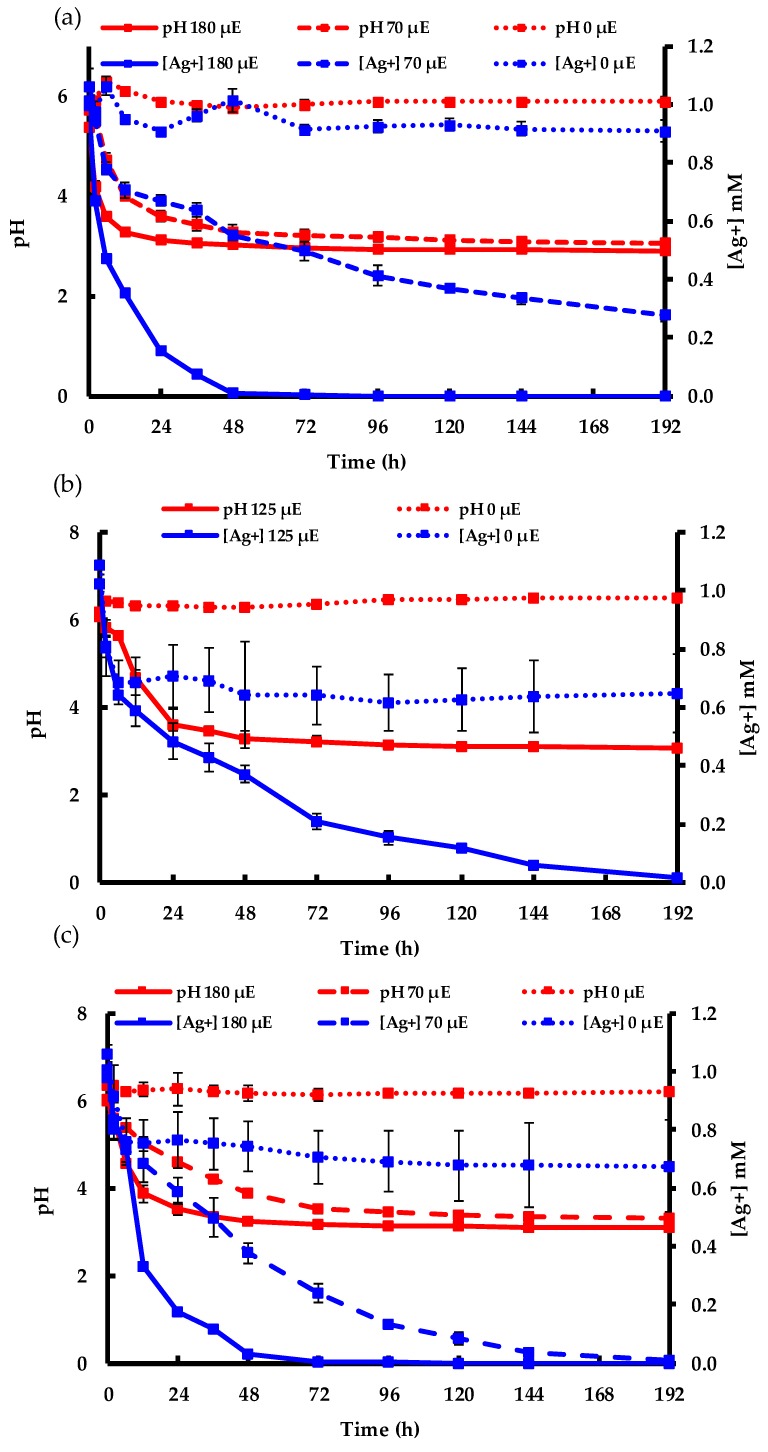
The change in pH and [Ag^+^] vs. time after Ag^+^ addition in the dark and under varying illumination intensities with EPS concentrations of: (**a**) 0.15 mg mL^−1^; (**b**) 0.38 mg mL^−1^; (**c**) 0.60 mg mL^−1^.

Figure 7 shows the change in pH and consumption of Ag^+^ in the factorial design experiment over 192 h. A significant difference in both pH and [Ag^+^] was found between the experiments conducted in light and dark conditions (Figure 7a–c). In the dark, no obvious change in pH was observed, corroborating the results discussed in Figure 1. Ag^+^ disappeared from the aqueous phase in the dark experiments, with a higher Ag^+^ removal rate at higher EPS concentrations (Figure 7b,c). The Ag cations were adsorbed by the EPS biomolecules and, consequently, could not be detected by the silver-ion-selective electrode. Although Ag^+^ adsorption occurred in the dark, the AgNP SPR absorption band was only observed in the presence of light, again verifying that photons are an obligate input for the AgNP synthesis reaction (Figure 5). More interestingly, at the same EPS concentration, the rate of decrease in both pH and [Ag^+^] was faster at higher light intensities than at lower intensities, indicating that the production kinetics were related to photon input (Figure 5b and Figure 7a,c). In addition, the acidification of the reaction mixture was measured by a change in pH from neutral (~7) to acidic (~3), indicating that during the synthesis process electrons were transferred to Ag^+^ to produce Ag^0^, with the charge balance being satisfied by the production of H^+^. However, the possible sources of H^+^ and its contribution in the biosynthesis process are further discussed in the following section.

**Figure 8:**
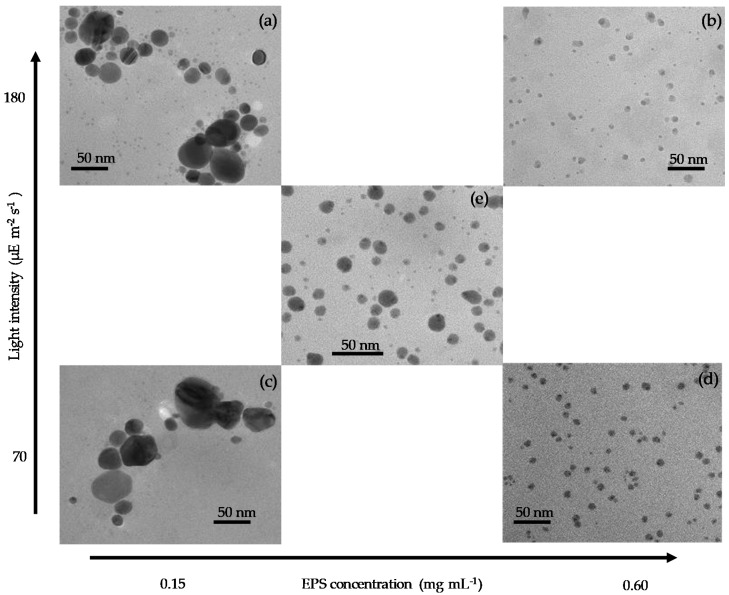
Transmission electron microscope (TEM) micrographs of AgNP samples: (**a**) EPS-0.15-1 mM-180 μE; (**b**) EPS-0.60-1 mM-180 μE; (**c**) EPS-0.15-1 mM-70 μE; (**d**) EPS-0.60-1 mM-70 μE; (**e**) EPS-0.38-1 mM-125 μE.

**Figure 9:**
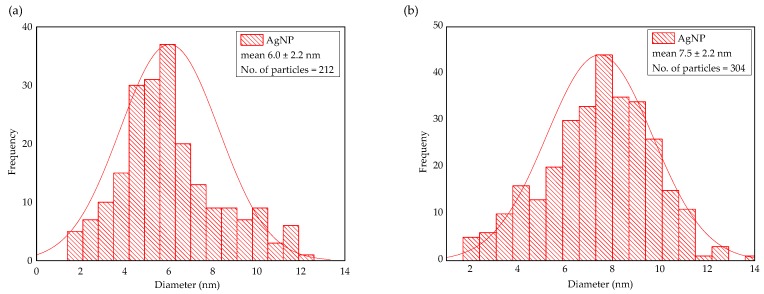
Particle size distribution of AgNPs for samples: (**a**) EPS-0.60-1 mM-180 μE; (**b**) EPS-0.60-1 mM-70 μE.

Figure 8 shows transmission electron microscope (TEM) micrographs of the AgNPs produced at various EPS concentrations and light intensities. A complete visualization of the five micrographs reveals that the AgNPs produced at lower EPS concentrations (Figure 8a,c) were significantly larger in size compared to the ones produced at higher concentrations (Figure 8b,d,e). This could be explained by the aggregation and sedimentation of unstable or partially stable particles at higher Ag^+^/EPS ratios. These observations corroborate the decrease in SPR peak intensity over time, as discussed earlier in Figure 2b, Figure 3b and Figure 5b. In contrast, lower Ag^+^/EPS ratio produced AgNPs that were well dispersed and mostly spherical in shape, as reported previously [13,25]. Furthermore, Figure 9 shows the particle size distribution of the selected samples at higher EPS concentrations. The average size of the AgNPs produced was 6.0 ± 2.2 nm (*n* = 212) for the EPS-0.60-1 mM-180 μE sample (Figure 9a), and 7.5 ± 2.2 nm (*n* = 304) for the EPS-0.60-1 mM-70 μE sample (Figure 9b). The fact that high amounts of EPS can promote the production of well-dispersed AgNPs has been discussed elaborately in a previous study [8].

### 2.4. The Sole Impact of Ag^+^ Adsorption on AgNP Production 

**Figure 10:**
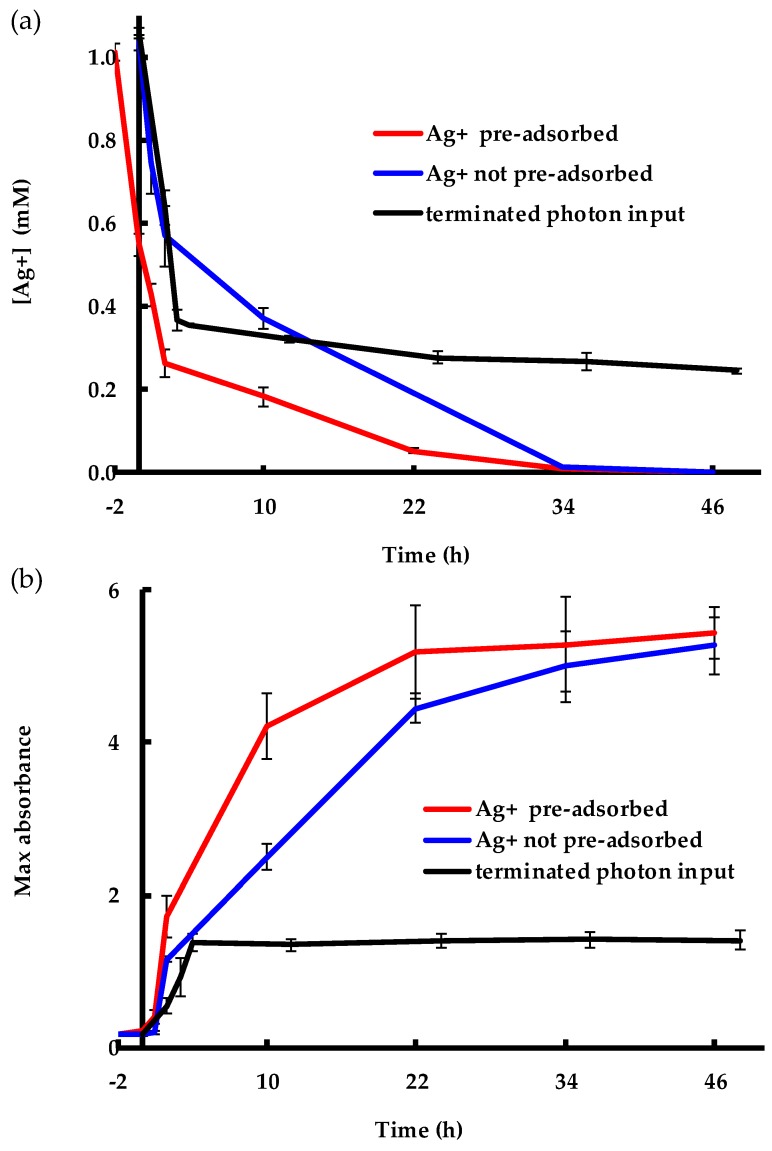
The results from the three experiments showing (**a**) consumption of Ag^+^ and (**b**) SPR development of the AgNPs over time.

Figure 10 shows the results from the three experiments where: (i) Ag^+^ was pre-adsorbed by the EPS that were challenged by AgNO_3_ in the dark for 2 h before the reaction mixture was exposed to light; (ii) Ag^+^ was not pre-adsorbed; and (iii) photon input was terminated after 2 h of synthesis (the detailed method is presented in the Materials and Methods section). Figure 10a shows the concentration of Ag^+^ in the aqueous phase while Figure 10b shows the corresponding development of the maximum SPR of AgNPs for the same experiments. Upon investigating both figures, it is worth noting that Ag^+^ adsorption prior to light exposure made the SPR band more intense compared to the process without pre-adsorption (red vs. blue curve in Figure 10b). With time, the two SPR curves converged to the same value (~5.4). As the AgNP formation continued, the Ag^+^ was continually removed from the aqueous phase while AgNP formation continued, which indicates a light-driven conversion of Ag^+^ to AgNPs. The Ag^+^ concentration curves (Figure 10a) of these two experiments eventually met at 34 h, but a dissimilar concentration curve was observed due to Ag^+^ adsorption within the first 2 hours for the pre-adsorbed case that made AgNP formation more apparent after the start of illumination, compared to the case where Ag^+^ was not allowed to adsorb before illumination. For the third experiment, Ag^+^ adsorption and AgNP formation occurred at the same time in the beginning; however, photon input was terminated at 2 h. Nevertheless, the AgNP formation continued for another couple of hours and, interestingly, the absorbance that could be attributed to AgNPs more than tripled after the termination. This phenomenon could be attributed to the continued growth of the as-produced particles before being capped by the EPS to terminate growth [17], therefore indicating that the stabilization process is photon-independent. In contrast, the adsorption of Ag^+^ continued at a very low rate compared to the other two experiments where AgNP formation occurred. In summary, the current results indicate that both Ag^+^ adsorption and AgNP stabilization do not depend on light, whereas Ag^+^ to Ag^0^ conversion does. These results corroborated the results discussed in Figure 1, Figure 2, Figure 3, Figure 4, Figure 5, Figure 6 and Figure 7. Moreover, these findings suggest that EPS play a direct role in the biosynthesis process by adsorbing Ag^+^.

### 2.5. Understanding the Possible Mechanism of Light-Dependent Biosynthesis of AgNPs

To understand the EPS-mediated, light-induced AgNP synthesis mechanism, it is crucial to identify the specific EPS biomolecules that can adsorb Ag^+^ and reduce them to Ag^0^ with a concomitant release of H^+^ into the reaction media. The major functional groups in *C. reinhardtii* EPS, measured by FTIR in our previous work, are presented in Table 1 [13]. These functional groups represent of polysaccharides, polyphenols and proteins, corroborating previous studies [26,27]. Table 1 shows the availability of functional groups known to donate electrons, such as OH^−^ [17,19,28], that reduce various metal ions to their corresponding metallic counterparts, as well as functional groups that can stabilize the NPs.

**Figure 11:**
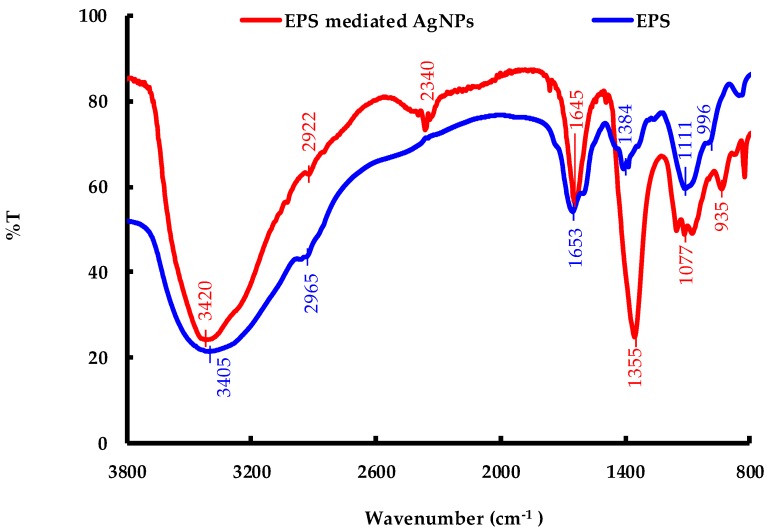
Fourier-transform infrared spectroscopy (FTIR) spectra of the EPS of *C. reinhardtii* and EPS-produced AgNPs.

Figure 11 presents the FTIR spectra of *C. reinhardtii* EPS and the AgNPs produced, and offers information regarding the shifts in the intensities and positions of the functional groups involved in the biosynthesis process [13,29]. The EPS-mediated AgNP sample showed an obvious increase in the transmittance at 3420 cm^−1^, 2340 cm^−1^, 1645 cm^−1^, 1355 cm^−1^, and 935 cm^−1^ compared to the EPS sample, which indicates a greater retention of functional groups such as OH^−^, ([NH]C=O), –COOH, and C–O–C in the former sample after the reduction occurred [21,30,31]. Additionally, these peaks became sharper after reduction, which indicates the consumption of the corresponding functional groups at these positions [31]. Besides the change in peak intensity, almost all the peaks shifted from their initial positions; in particular, the peaks at 2922 cm^−1^, 1355 cm^−1^, and 1077 cm^−1^ shifted significantly from their respective original positions at 2965 cm^−1^, 1384 cm^−1^, and 1111 cm^−1^. Overall, these spectroscopic changes confirmed that: (i) proteins and polysaccharides are mainly responsible for the reduction and stabilization of AgNPs and (ii) organic moieties are present on the AgNP surface [28,29,31].

Algal proteins are well known to reduce Ag^+^ and control AgNP shape [14,32,33]. For example, a previous study showed that amino acids, such as tyrosine, donated electrons to reduce Ag^+^ to produce AgNPs [14]. Moreover, the oxidation of the biomolecules is concomitant with the release of H^+^ into the reaction media, as observed in the current work, by the decrease in pH (Figure 7) [17]. 

Ag^+^ can also form a complex with amino acid residues, indicating that Ag^+^ adsorption to proteins in the current study could be responsible for the decrease in the measured Ag^+^ concentration in Figure 1. Sugars, also found in the EPS (Table 1), can be oxidized to sugar acids (e.g., glucose to glucuronic acid), which releases an electron that can reduce Ag^+^ to Ag^0^ [34]. Therefore, oxidation of diverse components of EPS under photon irradiation could explain how the EPS can increase the AgNP production with a consequent decrease in pH (Figure 7), as observed in the current study. Therefore, it is obvious that the decrease in pH is solely associated with the formation of AgNPs. Previous studies have reported the use of polysaccharides to produce AgNPs without the need to control the pH [35,36]. However, mild heating has usually been needed in these works, whereas the current method required light input. In fact, polysaccharides are known to act as both reducing agents and stabilizers. Therefore, polysaccharides in the EPS of *C. reinhardtii* can reduce Ag^+^ and cap nascent Ag^0^ to form stable AgNPs, as also reported previously [13,26]. In addition, it has been hypothesized that the photoexcitation of AgNP could catalyze the reaction between Ag^+^ and a carboxyl group through light-induced ligand rearrangement [37]. This hypothesis is supported by our results of Ag^+^ adsorption (Figure 7 and Figure 10a) and light-dependent Ag^0^ formation (Figure 3b and Figure 10b). Furthermore, our results showed that the stabilization of AgNPs is a function of EPS concentration. For example, Figure 5b shows that insufficient EPS led to partial aggregation and sedimentation of AgNPs and a decrease in the SPR band intensity. This fact is further corroborated by the TEM images presented in Figure 8a,c.

**Figure 12:**
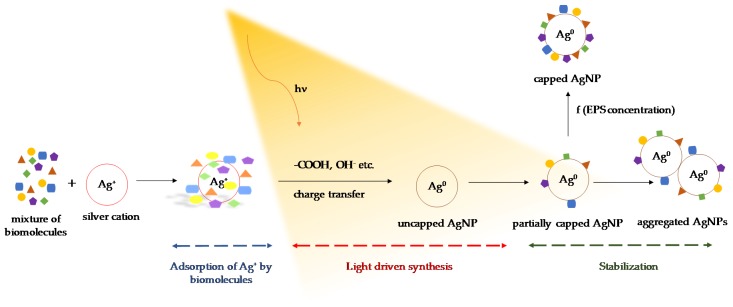
The proposed three-step mechanism of AgNP synthesis using EPS of *C. reinhardtii*.

The overall three-step mechanism is summarized in Figure 12. First, Ag^+^ is adsorbed to EPS biomolecules, as discussed above. In the second step, a light-dependent reaction occurs where functional groups within the EPS (Table 1) transfer electrons to Ag^+^ to form Ag^0^ and to facilitate the deposition of Ag^0^ until AgNPs are formed. Finally, EPS or components from EPS cap and stabilize the AgNPs to provide colloidal stability.

## 3. Materials and Methods

### 3.1. Cell Culture Maintenance and Monitoring

#### 3.1.1. Media Preparation

*C. reinhardtii* was cultured in a modified 3N-Bold’s Basal Medium (BBM)+V. The composition of the modified 3N-BBM+V medium was: 430 μmol L^−1^ K_2_HPO_4_, 1.3 mmol L^−1^ KH_2_PO_4_, 300 μmol L^−1^ MgSO_4_·7H_2_O, 2.94 mmol L^−1^ NaNO_3_, 128 μmol L^−1^ CaCl_2_·2H_2_O, 430 μmol L^−1^ NaCl, 132 μmol L^−1^ EDTA, 18 μmol L^−1^ FeSO_4_·7H_2_O, 185 μmol L^−1^ H_3_BO_3_, 4.91 μmol L^−1^ ZnCl_2_, 1.17 μmol L^−1^ MnCl_2_·4H_2_O, 1.01 μmol L^−1^ CuSO_4_·5H_2_O, 280 nmol L^−1^ CoCl_2_·6H_2_O, and 794 nmol L^−1^ Na_2_MoO_4_. All chemicals were of analytical grade and purchased from Sigma-Aldrich (St. Louis, MO, USA) or VWR (Radnor, PA, USA); deionized water (DIW) was the solvent. The average pH of the prepared BBM was 6.7 ± 0.2. The BBM was freshly prepared and sterilized by autoclaving at 121 °C and 1 atm gauge for 20 min, then allowed to cool for 24 h before being used for sub-culturing.

#### 3.1.2. Sub-Culturing of *C. reinhardtii*

*C. reinhardtii* strains were purchased from the *Chlamydomonas* Resource Center (University of Minnesota, St. Paul, MN, USA). Axenic sub-culturing was done every week in a Labconco Purifier Clean Bench (Labconco Corporation, Kansas City, MO, USA). All materials and BBM were autoclaved prior to use. New generations were prepared by adding 30 mL of the previous generation culture to 120 mL of BBM in a 500 mL borosilicate Erlenmeyer flask. The flasks were kept under an average illumination of 69 ± 5 μE m^−2^ s^−1^, provided by cool white LED tubes. The photoperiod was maintained at 16 h/8 h light/dark. The ambient temperature was maintained at 22 ± 1 °C.

### 3.2. Design of Experiments for Ag^+^ to AgNP Bioreduction Process

#### 3.2.1. Using OFAT Experimental Design to Study the Impact of Light on AgNP Biosynthesis Using EPS

For the OFAT (one-factor-at-a-time) experiment, the EPS supernatant was separated by centrifuging whole living cultures of *C. reinhardtii* at 3000× *g* for 5 min. Next, 50 mL of EPS were transferred to 250 mL Erlenmeyer flasks and AgNP synthesis was carried out by adding 5.6 mL of AgNO_3_ stock solution to achieve a working volume of 55.6 mL at concentrations of 0.125 mmol L^−1^, 0.625 mmol L^−1^, and 1.250 mmol L^−1^. The flasks were then kept in light or dark conditions. For the light condition, the average light intensity was maintained at 69 ± 5 µE m^−2^ s^−1^ and all flasks were separated by ~5 cm to eliminate any shading from one flask onto another. For the dark condition, the flasks were wrapped in a black cloth and kept out of any visible light source. Sampling was done aseptically with a 1 mL micropipette in a Labconco Purifier Clean Bench (Labconco Corporation, Kansas City, MO, USA).

All experiments were carried out in triplicate. To examine the impact of light, two out of the three replicates (L24+ and L48+) from each dark experiment were exposed to light after 24 h and 48 h, respectively, while keeping the third replicate (L-) in the dark as a control, as shown in Table 2.

In order to measure the exact amount of EPS put into each flask, the EPS extract was concentrated at 60 °C using an IKA RV 10 Rotary Evaporator (IKA Works, Inc., Wilmington, NC, USA). To remove the BBM salts, the concentrate was dialyzed against sterile DIW using Fisherbrand regenerated cellulose dialysis tubing with MWCO of 3500. Next, the retentate was freeze-dried for 24 h using the Labconco Freezone Freeze Dry System (Labconco Corporation, Kansas City, MO, USA) to obtain a dry biomass. The measured concentration of EPS in each flask was ~0.46 mg mL^−1^.

#### 3.2.2. Using Factorial Experiment Design to Study the Impact of Both Light and EPS on AgNP Biosynthesis

For the factorial design method, a 2^2^ (2 level 2 factor) factorial design was adopted [38]. Light intensity and EPS concentrations were used as two factors at low (−) and high (+) levels, as presented in Table 3. The conversion from coded to real world value is given in Equation (1).

(1) Coded value=(Real value)−(Center value)12(Range)

The center value was determined by averaging the real values.

To carry out the experiment, the EPS supernatant was first concentrated, dialyzed to remove BBM salt, and freeze-dried to obtain a dry biomass, following the procedure described above (Section 3.2.1). Finally, dried EPS was dissolved in sterile DIW in different amounts to achieve different concentrations of EPS, according to the design of the experiment (Table 3). These experiments were conducted in petri dishes (8.5 cm diameter) instead of Erlenmeyer flasks (cf. OFAT experimental design) to ensure more homogeneous illumination. The working volume of each dish was 30 mL, with a reaction mixture depth maintained at ~0.5 cm. Each experiment was carried out in triplicate at AgNO_3_ concentration of 1 mmol L^−1^. The same experiments were carried out in the dark as control experiments. In addition, one experiment was done with EPS but without AgNO_3_ in light.

Three additional experiments were carried out in order to further understand the photon-induced synthesis and the role played by the EPS. These experiments were also conducted in petri dishes with the same working volume as reported above; however, fixed EPS concentration and light intensity were used. The freeze-dried EPS, as detailed above, was dissolved in sterile DIW to achieve a final EPS concentration of 0.60 mg mL^−1^ in each experiment, as shown in Table 4 and Table 5. Out of the three experiments, two experiments started at −2 h; Experiment 1 containing EPS and 1 mM AgNO_3_ and Experiment 2 containing only EPS were kept in the dark for 2 h before being exposed to light (Table 4). Each experiment was carried out in triplicate and petri dishes were placed at random positions on the shelf. Table 4 shows the design of experiment at t = −2 h.

After 2 h (at t = 0 h), Experiments 1 and 2 were both exposed to light, as shown in Table 5. Additionally, AgNO_3_ was added to Experiment 2 so the final concentration was 1 mM, the same as in Experiment 1. At the same time, Experiment 3 started when 0.60 mg mL^−1^ EPS with 1 mM AgNO_3_ were exposed to light. However, this experiment was moved into the dark after 2 h (t = 2 h) while Experiments 1 and 2 continued in the light.

### 3.3. Characterization Techniques

#### 3.3.1. Spectrophotometric Characterization

The spectrophotometric characterization was performed using a Cary-100 Bio UV-Vis Spectrophotometer (Agilent Technologies, Santa Clara, CA, USA). Deionized water was used as the blank and all the analyses were carried out in 1.00 cm path length polystyrene cuvettes. The samples were scanned from 380 nm to 800 nm. The crude reaction samples saturated the spectrophotometer, so the samples were diluted 10× with deionized water, and their UV–vis spectra were recorded, multiplied by 10, and reported in the current study. To study the shift in the SPR peak, the difference between the maximum peak absorbance at a particular wavelength (λ_max_) and the absorbance at 800 nm (λ_800_) was determined and plotted vs. time.

#### 3.3.2. Determination of Ag^+^ Concentration

The consumption of Ag^+^ was determined by measuring the free Ag^+^ ion concentration with a silver-ion-selective electrode (ISE) (Hanna Instruments, Woonsocket, RI, USA). The electrode was calibrated before measuring each experiment using 0.125 mmol L^−1^, 1.250 mmol L^−1^, and 12.500 mmol L^−1^ AgNO_3_ solutions.

#### 3.3.3. Determination of pH

The pH was measured using an Oakton pH meter (Oakton Instruments, Vernon Hills, IL, USA).

#### 3.3.4. Morphological Analyses

Transmission electron microscopy (TEM, JEOL Ltd., Tokyo, Japan) was used to characterize AgNP morphology. Samples were prepared using a method reported previously by Rahman et al. [13]. The reaction media was first filtered using a glass microfiber filter (diameter 25 mm, pore size 1.2 µm). TEM samples were prepared by casting 30 μL of filtrate onto the surface of a PELCO^®^ (Fresno, CA, USA) TEM Grid Carbon Type- B (Ted Pella Inc., 3.05 mm O.D., 400 mesh, 0.4 × 2 mm single slot Cu) and air-dried for 24 h. The TEM analysis used a JOEL JEM-1400 Plus Transmission Electron Microscope (120 kV, 1 kV step, 69 μA beam current, 100 k× magnification, spot size 1) equipped with embedded scanning transmission electron microscopy (STEM, JEOL Ltd., Tokyo, Japan).

#### 3.3.5. Fourier-Transform Infrared Spectroscopy (FTIR)

FTIR analyses of the EPS and EPS-mediated AgNPs were carried out using the potassium bromide (KBr) pellet method, as reported previously by Rahman et al. [13]. The samples were first centrifuged at 6000× *g* for 10 min, and the supernatant was filtered using a glass microfiber filter (diameter 25 mm, pore size 1.2 µm). The filtration step was repeated twice to ensure the removal of uncoordinated biological materials. The samples were then freeze-dried using a Labconco Freezone Freeze Dry System (Labconco Corporation, Kansas City, MO, USA). Finally, 4 mg of the dried samples were mixed with KBr in a 1:50 ratio to prepare the pellet. The pellets were kept in an air-tight container at room temperature until measurement to minimize the amount of bound water. The FTIR spectra of the pellets were recorded using a Thermo Scientific Nicolet iS10 FTIR Spectrometer (Thermo-Scientific, Waltham, MA, USA). The machine operated in transmittance mode at a resolution of 4 cm^−1^, each pellet was scanned in the spectral range of 4000–800 cm^−1^, and the results are presented in percent transmittance (%T).

### 3.4. Statistical Techniques

All the experiments were carried out in triplicate and averaged data are presented with respective error bars equal to one standard deviation. The standard deviations were calculated using the Microsoft Excel software program (version 16.0, Redmond, WA, USA). For particle size distribution analyses, frequency histograms were plotted from the raw particle size data obtained using ImageJ software (version 1.8.0, Bethesda, MD, USA), developed by the National Institutes of Health (NIH), Bethesda, MD, USA. The following equation was used to calculate the bin width for the histograms, where N is the square root of the number of data values:

(2) $$\mathrm{Bin}\;\mathrm{width}\;=\;\frac{\mathrm{maximum}\;\mathrm{value}\;-\;\mathrm{minimum}\;\mathrm{value}}{\mathrm{number}\;\mathrm{of}\;\mathrm{bins}\;(N)}$$

Furthermore, the histograms were curve-fitted using the Gaussian peak function in OriginPro software (version 9.0, Northampton, MA, USA).

## 4. Conclusions and Future Perspectives

The current study proposed a light-driven AgNP synthesis mechanism using EPS of *C. reinhardtii*. In this mechanism, Ag^+^ is first adsorbed by EPS biomolecules while a light-driven reaction reduced Ag^+^ to AgNPs. Although the adsorption of Ag^+^ occurred with and without photons, the EPS-mediated AgNP formation was exclusively light-driven. The production of H^+^ during AgNP synthesis led to the acidification of the reaction mixture. More interestingly, the pH and Ag^+^ were found to concomitantly decrease in the reaction mixture, and the rate of decrease was boosted as light intensity increased. The kinetics and yield of AgNP synthesis were dependent on both EPS concentration and light intensity. Additionally, several functional groups, including –COOH, OH^−^, and C–O–C from EPS biomolecules, have been suggested to be contributors to the conversion of Ag^+^ to Ag^0^ and the stabilization of the as-produced AgNPs. It was also demonstrated that as EPS concentration was reduced, the stability of the AgNPs in solution was also reduced. Moreover, the stabilization process was found to occur independently of photon input. The most significant discoveries from the current study include: (i) a direct role played by the EPS by adsorbing Ag^+^ and (ii) the constitutive relationship between the light intensity, EPS concentration, and biosynthesis of AgNPs. However, further characterization of the EPS and AgNPs is required to shed light on this process. In addition, adsorption coefficient and the conversion of adsorbed Ag^+^ to AgNPs need to be determined in order to develop scalable PBR and EPS–PBR–wastewater systems.

## Figures and Tables

**Table 1:** Biomolecules and functional groups in EPS and their role in AgNP biosynthesis.

Biomolecules	Wavenumbers	Functional Groups	Observation	Reference
Proteins	1640 cm^−1^	OH^−^, ([NH]C=O), –COOH	Ag^+^ reduced and shape controlled AgNPs stabilized	[14,19]
Polysaccharides	3400 cm^−1^, 1355 cm^−1^, 935 cm^−1^	OH^−^, C–O–C, COO^−^	Ag^+^/Au^+^ reduced and NPs stabilized	[19,28]
Polyphenols	3400 cm^−1^, 1075 cm^−1^	OH^−^, C–O	Ag^+^/Au^+^ reduced and NPs stabilized	[19,28]
*C. reinhardtii* EPS	3400 cm^−1^, 1640 cm^−1^, 1075 cm^−1^, 935 cm^−1^	OH^−^, ([NH]C=O), C–O, C–O–C,C–O–H, COO^−^	Ag^+^ reduced and AgNPs stabilized	[13]

**Table 2:** The one-factor-at-a-time (OFAT) experiment design at three AgNO_3_ concentrations.

Dark Experiments	Replicates	Description
D-0.125 mM	L24+	Exposed to light after 24 h
	L48+	Exposed to light after 48 h
	L-	Not exposed to light
D-0.625 mM	L24+	Exposed to light after 24 h
	L48+	Exposed to light after 48 h
	L-	Not exposed to light
D-1.250 mM	L24+	Exposed to light after 24 h
	L48+	Exposed to light after 48 h
	L-	Not exposed to light

**Table 3:** The factorial design of experiment.

Level	Light Intensity (µE m^−2^ s^−1^)	EPS Concentration (mg mL^−1^)
low (−)	70	0.15
high (+)	180	0.60
mid	125	0.38

**Table 4:** The design of experiment starting at t = −2 h.

t = −2 h			
Experiment	EPS Concentration (mg mL^−1^)	AgNO_3_ Concentration (mM)	Light Intensity (µE m^−2^ s^−1^)
Experiment 1	0.60	1	0
Experiment 2	0.60	0	0

**Table 5:** The exposure of Experiment 1 and Experiment 2 to light, and the start of Experiment 3 at t = 0 h.

t = 0 h			
Experiment	EPS Concentration (mg mL^−1^)	AgNO_3_ Concentration (mM)	Light Intensity (µE m^−2^ s^−1^)
Experiment 1	0.60	1	180
Experiment 2	0.60	1	180
Experiment 3*	0.60	1	180

* moved into the dark at t = 2 h.
